# Natural history of ROHHAD syndrome: development of severe insulin resistance and fatty liver disease over time

**DOI:** 10.1186/s40842-019-0082-y

**Published:** 2019-07-09

**Authors:** Abdel Wahab Jalal Eldin, Dilara Tombayoglu, Laura Butz, Alison Affinati, Rasimcan Meral, Mehmet Selman Ontan, Kelly Walkovich, Maria Westerhoff, Jeffrey W. Innis, Neehar D. Parikh, Elif A. Oral

**Affiliations:** 1 Division of Metabolism, Endocrinology and Diabetes (MEND), Department of Internal Medicine Brehm Center for Diabetes, Michigan Medicine, University of Michigan, 1000 Wall Street, Room 5313, Ann Arbor, MI 48105 USA; 2 Istanbul Faculty of Medicine, Istanbul University, Istanbul, Turkey; 3 Division of Pediatric Hematology/Oncology, Department of Pediatrics and Communicable Diseases, Michigan Medicine, University of Michigan, Ann Arbor, MI USA; 4 Department of Pathology, Michigan Medicine, University of Michigan, Ann Arbor, MI USA; 5 Division of Genetics, Metabolism and Genomic Medicine, Department of Pediatrics,and Departments of Human Genetics and Internal Medicine, Michigan Medicine, University of Michigan, Ann Arbor, MI USA; 6 Division of Gastroenterology and Hepatology, Department of Internal Medicine, Michigan Medicine, University of Michigan, Ann Arbor, MI USA

**Keywords:** ROHHAD, Insulin resistance, GLP-1 agonists, Obesity, Atypical diabetes

## Abstract

**Background:**

Rapid-onset obesity with hypothalamic dysfunction, hypoventilation, and autonomic dysregulation (ROHHAD) is a rare syndrome with unknown etiology. Metabolic abnormalities are not known to be part of the syndrome. We present one of the oldest cases reported in the literature, who developed severe metabolic abnormalities and hepatic disease suggesting that these features may be part of the syndrome.

**Case presentation:**

A 27-year-old woman, diagnosed with ROHHAD syndrome at age 15, who previously developed diabetes insipidus, growth hormone deficiency, hyperprolactinemia, and hypothyroidism in her first decade of life. This was followed by insulin resistance, NAFLD, liver fibrosis, and splenomegaly before age 14 years. Her regimen included a short course of growth hormone, and cyclic estrogen and progesterone. Her metabolic deterioration continued despite treatment with metformin. Interestingly, she had a favorable response to liraglutide therapy despite having a centrally mediated cause for her obesity. At age 26, a 1.6 cm lesion was found incidentally in her liver. Liver biopsy showed hepatocellular carcinoma which was successfully treated with radiofrequency ablation.

**Conclusion:**

Metabolic abnormalities, Insulin resistance and fatty liver disease are potentially part of the ROHHAD syndrome that may develop over time. GLP1 agonists were reasonably effective to treat insulin resistance and hyperphagia. Patients with ROHHAD may benefit from close follow up in regards to liver disease.

**Electronic supplementary material:**

The online version of this article (10.1186/s40842-019-0082-y) contains supplementary material, which is available to authorized users.

Rapid-onset obesity with hypothalamic dysfunction, hypoventilation, and autonomic dysregulation (ROHHAD) is a rare syndrome characterized by hyperphagia and rapid-onset weight gain that starts in early childhood. The rapid weight gain is followed by hypothalamic manifestations with neuroendocrine deficiencies, hypoventilatory breathing abnormalities, and autonomic dysregulation [1]. A genetic basis for ROHHAD has not been established, which differentiates it from central hypoventilation syndrome caused by mutations in the *PHOX2B* gene [2]. Natural history information about this rare syndrome is sparse, as the reported mortality rates are high at 50 to 60% [3] within a short period of time after diagnosis, usually due to hypoventilation, cardiopulmonary failure or both [4–6].

We have followed an unusual case of ROHHAD syndrome for two decades, currently age 27 years, and is believed to be one of the oldest patients with ROHHAD syndrome. Over time, she developed novel clinical features not previously associated with ROHHAD, such as severe insulin resistance and diabetes mellitus, severe hypertriglyceridemia, progressive fatty liver disease, and more recently clinical cirrhosis and hepatocellular carcinoma. The clinical management of the hyperglycemia has been challenging due to the severe insulin resistance and labile sodium and fluid balance.

## Methods

### Case report

This is a report of a patient who was followed at the Michigan Medicine Pediatric Endocrinology Clinic until age 18 years. She was also evaluated at the Lurie Children’s Hospital Autonomic Dysfunction Program at the Lurie Children’s Hospital of Chicago at age 16 years. Subsequently, endocrine care was transitioned to the Metabolism, Endocrinology and Diabetes Clinic Atypical Diabetes Program at Michigan Medicine. She was also followed by the Medical Genetics specialty clinic at Mott Children’s Hospital of Michigan Medicine during this time.

The patient gave consent to share her case presentation in the medical literature and provided her pictures. Laboratory testing was performed using standard procedures at the clinical pathology laboratory of the Michigan Medicine. *PHOX2B* gene testing was performed at the University of Chicago Genetics Laboratory and whole exome sequencing of the patient and her parents were performed by GeneDx (Gaithersburg, Maryland) using samples drawn in February 2017.

Hormonal measurements were performed using the standard procedures available in the clinical laboratory at Michigan Medicine manual available at the University of Michigan Department of Pathology [7] and were reported in the medical record. All the testing that is being reported here was accomplished during routine clinical care and no tests done for research purposes are being reported.

### Literature review

A Pubmed search conducted using the terms ROHHAD, ROHHADNET and the syndrome full name, retrieved 50 articles. These were reviewed and categorized in Additional file 1: Table S2 and further summarized after the case presentation. Prior to the literature search, we sought to investigate the age of onset, the life span, current ages of surviving cases and the frequency of metabolic (glucose intolerance, insulin resistance or presence of diabetes) as well as liver related complications from the reported cases.

## Case presentation

**Fig. 1:**
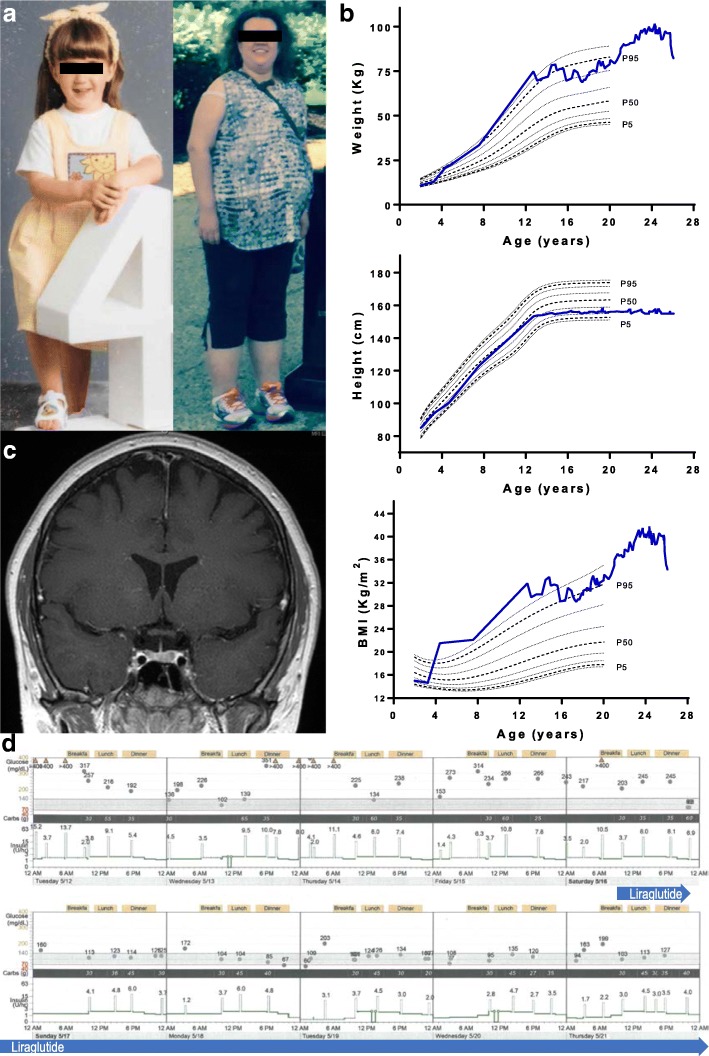
**a** Photograph of the patient at age 4 (left) compared to 26 (right). **b** Coronal view of brain MRI with no abnormality detected **c** Growth charts constructed with standard data obtained from CDC [20]. Stature (top), and weight (middle) display a drop in linear growth and significant weight gain at 4 years of age; increased linear growth upon initiation of growth hormone at age 7 with a plateau following discontinuation at age 14. BMI (bottom) has been in the obese range since age 4. **d** 10-day glucose tracing by insulin pump shows improvement in blood glucose after 8–12 h of starting GLP-1 agonist, liraglutide, on May 16

A previously healthy, normally developing girl without familial obesity was evaluated at age 4 years for a sudden weight gain of 15 pounds in less than 1 year (25th to 95th percentile for weight) and decreased linear growth (25th to 10th percentile for height; Fig. 1b). She displayed no features of lipodystrophy. The medical history of the patient revealed that she was born to non-consanguineous healthy parents with a birth weight of 4 pounds 13 oz (2.14 Kg) by c-section at 37 weeks (3 weeks preterm) due to pre-eclampsia. Her growth in the first year of life included a height at the 75th percentile and weight at the 25th percentile. She never had any concerns about developmental delays or cognitive impairment. However, her parents noted fear and anxiety about routine daily activities, entering crowded spaces as well as decision making, starting around age 4 years. Ophthalmic exam did not show any vision abnormalities other than myopia and astigmatism. Given her mother’s history of hearing impairment, hearing test was done but did not show any abnormality.

During the initial evaluation, her sodium was 156 mEq/L with undetectable antidiuretic hormone (ADH), consistent with diabetes insipidus and disrupted thirst regulation. She was also found to have central hypothyroidism, elevated prolactin, and growth hormone deficiency (Table 1). Her baseline cortisol levels were normal to high with normal ACTH. A hypothalamic tumor was suspected, but brain MRI was normal (Fig. 1c). Due to the rapid weight gain, she was also worked up for Cushing’s syndrome, however, abdominal CT revealed no adrenal masses and overnight dexamethasone suppression test was normal.

**Table 1:** Childhood labs

	Value	Reference Range
IGF-1, ng/mL	41	70–288
GH stimulation testing	GH undetectable at baseline and all-time points after stimulation with arginine and insulin-induced hypoglycemia to 49 mg/dL	
Prolactin, ng/mL	50	1–17
ADH, pg/mL	1.7	< 1.7
Serum Na, meq/L	156	132–145
Morning cortisol, mcg/dL	12.7	< 20
TSH, mU/L	0.87	0.3–5.5
Free T4, ng/dL	0.99	0.73–1.79
Estradiol at age 12, pg/mL	8.0	< 10 prepubertal
FSH, mIU/L	0.2	3–26
LH, mIU/L	0.5	2–105

Abbreviations: IGF-1, insulin like growth factor-1; GH, growth hormone; TSH, thyroid stimulating hormone; LH, luteinizing hormone. A leptin level at age 22 was measured at 99 ng/ml and an adiponectin level was 3 mcg/ml which was low

She was originally started on subcutaneous DDAVP, and then transitioned to intranasal spray. She was very closely monitored with weekly blood draws, tight fluid instructions and close weight tracking. Despite this tight monitoring, she still required hospitalizations for hypernatremia at the time of infections. Given these difficulties, she never tried oral DDAVP. She was simultaneously treated with levothyroxine replacement.

After age 10, she was diagnosed with nonalcoholic fatty liver disease (NAFLD) with evidence of progressive fibrosis over time, resulting in hypersplenism and pancytopenia. Her liver function tests (LFTs) were noted to be elevated starting at age 10. At age 11, she underwent a liver US showing the presence of increased fat as well as splenomegaly. During the same period, she was noted to have autonomic dysfunction, abnormal thermoregulation, and profuse sweating.

She was treated with growth hormone from age 10 to age 14 years to assist with linear growth and it was discontinued due to increased swelling and weight gain. At age 10, she was diagnosed with sleep apnea and insulin resistance. She was started on metformin, but developed clinical diabetes by age 14. She did not achieve spontaneous menarche despite the development of oily skin and acne and was started on female hormone replacement with cyclic estrogen and progesterone therapy at the age of 14.

At age 15, she developed respiratory failure and shock from an upper respiratory tract infection, requiring intubation, oscillator, and vasopressors. She was able to recover after a prolonged hospitalization. Shortly after at age 16, she underwent a thorough evaluation at the Lurie Children’s Hospital that documented her autonomic dysfunction and hypoventilation (Additional file 1: Table S1). Management with overnight ventilator, oxygen supplementation and oscillator use were recommended. Home continuous overnight monitoring for oxygen saturation and heart rate were also initiated. At that time, she underwent *PHOX2B* gene testing due to the presence of autonomic dysfunction and this testing was negative.

Between the ages of 18–23, she was transitioned to adult endocrine care where her diabetes control was noted to become more challenging. She was started on insulin, with insulin requirement increasing to > 310 international units (IU) while her HbA1c increased to numbers above 9%. Poor diabetes control made management of sodium balance very difficult due to polyuria and absence of thirst. She developed severe acanthosis nigricans and lymphedema. Her weight increased to BMI over 40 kg/m2. Over time, she was started on U500 insulin. She also developed hypertriglyceridemia at this time with the highest value at 1062 mg/dL. Of note, given the hypertriglyceridemia, low adiponectin levels and the lack of a commercial test, she was never tested for presence of the insulin receptor auto-antibody.

She was started on liraglutide 0.6 mg daily with the hope of improving control and sparing insulin to improve acanthosis nigricans, hyperandrogenism, weight and fatty liver. Her insulin requirements dropped precipitously to less than 30% of baseline doses within 8 to 12 h of initiation (Fig. 1d). She was managed with liraglutide 1.2 mg daily on and off for about 2 years. Initiation of liraglutide did not cause worsening of her diabetes insipidus, hypernatremia, or pancytopenia. She did, however, experience worsened diarrhea due to the use of liraglutide leading to treatment interruption. Her acanthosis nigricans, facial acne and coarsening of facial features improved due to dose reduction in insulin.

**Fig. 2:**
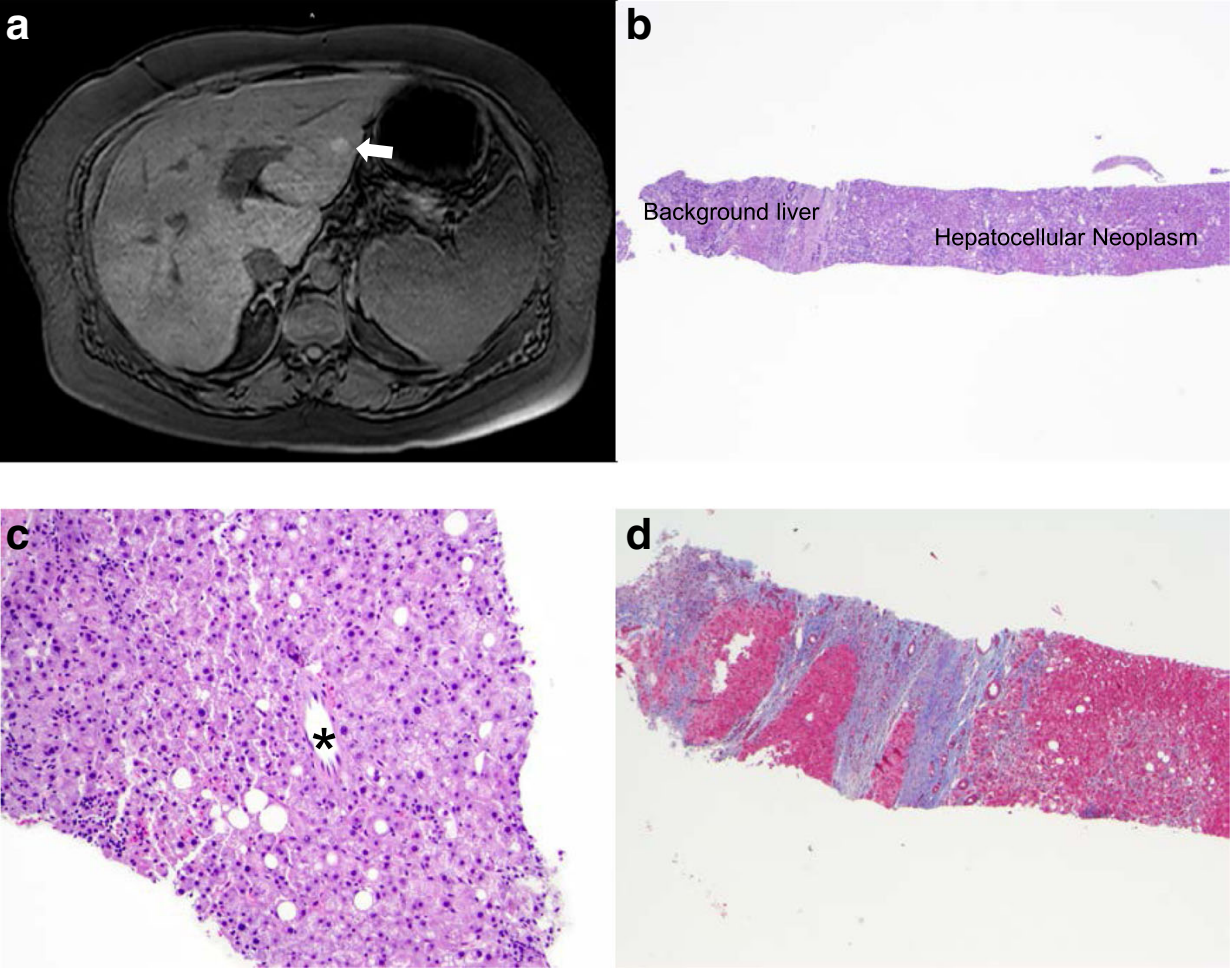
**a** MRI abdomen showing hepatocellular carcinoma in the left lobe (BCLC stage A). **b** The liver mass biopsy showed a small amount of fibrotic background liver. The main lesion was a well-differentiated hepatocellular neoplasm composed of sheets of lesional hepatocytes and absence of normal portal structures. **c** This higher magnification of the hepatocellular neoplasm shows an unpaired artery (*) and surrounding well-differentiated neoplastic hepatocytes. **d** Trichrome stain highlights cirrhotic background

With increased weight gain, swelling, worsening acanthosis nigricans, a weight reduction diet was attempted with meal replacement and caloric restriction. She lost 15 lbs. with this attempt. At the age of 26, she was noted to have a 1.6 cm Barcelona Clinic Liver Cancer (BCLC) Stage 0 (T1N0M0 Stage 1) liver lesion on routine semi-annual liver surveillance because of the presence of clinical cirrhosis and hypersplenism. The biopsy of the lesion favored the diagnosis of a well differentiated hepatocellular carcinoma (Fig. 2). A radiofrequency ablation procedure for this was successfully completed on 8/30/2018.

In addition, other comorbidities that developed over the years include cholelithiasis, chronic kidney disease with nephrotic range proteinuria (no biopsy performed to date to determine the underlying pathological mechanisms but being contemplated), hypertension, anemia and thrombocytopenia, splenomegaly, irritable bowel syndrome, vitamin D deficiency, and mild tricuspid regurgitation.

## Results of literature review

The supplemental file summarizes all cases documented in the literature. As shown, most cases were quite young at the time of death or last encounter before the report. Table 2 summarizes basic clinical characteristics of the cases and the presence of the features related to insulin resistance. Overall, there were 117 cases reported. Insulin resistance were documented in only 5 patients and none of them were older than teenage years. Fatty liver disease or nonalcoholic steatohepatitis has not been reported previously. Definitive deaths of only 8 patients were documented in the papers even though many of the reviews on this disease entity report a high incidence of mortality before teenage years. Cause of death was predominantly cardiopulmonary, but infections were also reported to lead to death. Of note and not included on the table, premature birth and low birth weight are not typical features of ROHHAD.

**Table 2:** Case summary

	Result
Diagnosis age, years (*n* = 23)	5.6 (3.1)
Age at last encounter, years (*n* = 25)	8.9 (4.7)
Number of cases reported	117
Females, male	15, 34
Hypercholesterolemia	4
Insulin resistance	5
Reported deaths	8
Case of death	
Cardiorespiratory arrest	4
Respiratory pathology	1
Pneumonia	1
Septic shock	1
Disconnection from ventilator support	1

Data are presented as Mean (SD) or count out of 117 cases.

## Discussion

Our patient is one of the oldest described with ROHHAD who has developed severe insulin resistance while under observation. This case expands the spectrum of clinical findings in ROHHAD, to include severe insulin resistance progressing to diabetes mellitus with complications, progressive NAFLD with hypersplenism, and cirrhosis as well as hepatocellular carcinoma.

ROHHAD syndrome is a relatively novel disease. It was first described by Fishman et al. in 1965 [8], but the syndrome acquired the name in 2007 by Ize-Ludlow et al. when the diagnostic criteria were described [9]. To date, more than 100 cases (*n* = 117) based on our review) have been reported in the literature [5]. Recently Lee et al. reported a systematic review of the reported cases independent of our efforts, but this review did not focus on insulin resistance, dyslipidemia or fatty liver disease. In fact, the summary tables found in that review do not comment specifically on any of these novel features [10]. The syndrome is quite distinctive from Prader-Willi and other complex syndromes of obesity due to its sudden onset acquired nature, absence of symptoms at birth, predominance of autonomic features, and respiratory abnormalities as well as diabetes insipidus and hyperprolactinemia.

The condition is characterized by high morbidity and mortality rates [3, 11]. Forty per cent of patients were noted to develop neural crest tumors (ganglioneuroma, ganglioneuroblastoma); it was suggested that the acronym ROHHAD could be extended to ROHHADNET (ROHHAD-neuroendocrine tumors) [11]. Previously recognized features of the syndrome include obesity, alveolar hypoventilation, autonomic dysregulation, sympathetic nervous system tumors. While at this point, we cannot rule out the existence of two separate disorders in our patient leading to a blended phenotype, we suggest that severe insulin resistance and progressive fatty liver disease may be features to the syndrome. We also want to draw attention to this case as a cause for the development of atypical diabetes. Evaluation of patients with ROHHAD shoudl include evaluation for these potential complications and with time, we may learn the frequency of these features in ROHHAD. 

The etiology of the syndrome is not known. Molecular diagnostic studies including *PHOX2B* gene testing and whole exome interrogation in conjunction with her parents did not reveal any causative genetic alterations in our patient including monogenic or syndromic obesity or known syndromes of insulin resistance (including the insulin receptor). In 2011, Patwari et al. reported monozygotic twins, one of whom had ROHHAD syndrome while the other twin did not, [12] suggesting that the disease may not develop due to genomic changes. Even though there is no definitive understanding, it is postulated that ROHHAD may be an immune-mediated central nervous system (CNS) disorder [6, 13–15] or epigenetic factors may have a role in the pathogenesis of the syndrome [12]. In this regard, it was interesting that our patient did not demonstrate any laboratory features suggesting other autoimmune diseases at any point. Periodically performed imaging studies did not reveal any neural crest tumors to date either.

Other than the well described pituitary hormonal deficiencies, diabetes insipidus and hyperprolactinemia, other hormonal features of this syndrome are not described. Our patient had a high circulating leptin level. We are not sure if this can be generalized to other ROHHAD patients. We did not study the incretin secretion or other circulating mediators of appetite control or energy balance in our patient and are not aware of these investigations in other patients.

Our case presented several challenges and lessons to us that are worth sharing. She was prepubertal when she presented with the earliest signs of insulin resistance. She was treated with metformin almost immediately, but this did not prevent the development of severe acanthosis nigricans, oily skin, hirsutism and coarsening of features, despite the presence of central hypogonadism, suggesting that insulin resistance alone can drive these aspects of clinical presentation without any central stimulation of the pituitary. She was treated with cyclic estrogen and progesterone replacement for bone health. Our patient was also treated with growth hormone for several years to assist with linear growth, but this was kept short due to worsening weight gain and swelling. Management of her case from childhood through early adulthood remained quite complicated and she required frequent lab monitoring and clinical visits. Both her parents and herself remained very vigilant about her care. Long term survivors with this syndrome need to be closely monitored and care transitions from pediatric to adult endocrinology should be undertaken in a planned manner.

Insulin resistance and adult growth hormone deficiency are associated with non-alcoholic fatty liver disease (NAFLD) and non-alcoholic steatohepatosis (NASH); the latter can progress to liver cirrhosis and hepatocellular carcinoma in some cases [16]. High doses of insulin, growth hormone, and even estrogen may have also contributed to the development of hepatocellular cancer [17]. In addition, prolactin receptors are sometimes expressed in hepatocellular cancers [18] and our patient has had mild, but untreated hyperprolactinemia since the onset of her condition. Our patient’s hyperprolactinemia was never adequately addressed; dopamine agonists were never initiated due to the presence of autonomic dysfunction, and fear of worsening orthostasis. We recommend screening for hepatic lesions using abdominal ultrasound in other patients presenting with the clinical features of NAFLD in the setting of ROHHAD as they grow older, particularly if they have signs of advanced hepatic fibrosis or cirrhosis. In addition, presence of clinical diabetes and insulin resistance should also be screened in addition to various endocrine manifestations.

The immediate improvement in glycemic control following liraglutide initiation indicates that the insulin resistance seen in this patient can be partially overcome with glucagon-like peptide-1 (GLP-1) agonism and adds to the growing evidence that GLP-1 receptor agonists may exert their metabolic actions via neural circuitry located outside of the hypothalamus, or via peripheral mechanisms [19]. Treatment experience in a larger number of patients is needed to validate these early observations.

Finally, our patient’s sodium and fluid balance management were markedly tenuous on account of poorly controlled diabetes, presence of diabetes insipidus and absence of thirst regulation. The patient required weekly sodium monitoring and constant adjustment of her fluid intake recommendation and her DDAVP dose. Inter-current illnesses (such as upper respiratory tract, urinary tract infections, or cellulitis episodes) and natural temperature fluctuations impacted her sodium levels. Our patient had multiple hospitalizations for dehydration and hypernatremia despite being compliant with instructions and carrying out frequent laboratory testing. Ideal glucose control (also a challenging goal for this patient) was essential to maintain her sodium balance closer to the normal range.

## Conclusion

ROHHAD is an interesting syndrome presenting with a characteristic cluster of abnormalities. There appears to be heterogeneity among patients diagnosed with ROHHAD and careful clinical characterization is required to more precisely define this disease entity. Our case expands on the clinical features of this syndrome to include severe and progressive insulin resistance and fatty liver disease. It should be noted that the fatty liver disease in this condition can be aggressive and result in development of cirrhosis that can be complicated by hepatocellular carcinoma.

## Additional file

Additional file 1: **Table S1.** Autonomic testing available on subject. **Table S2** Summary data abstracted from reported cases and respective references. (DOCX 79 kb)

## Data Availability

All data analyzed during this study are included in this published article.

## Abbreviations

ADH: Antidiuretic hormone
BCLC: Barcelona Clinic Liver Cancer
DDAVP: Desmopressin Acetate
GLP-1: Glucagon-like peptide 1
LFT: Liver function test
NAFLD: Non-alcoholic fatty liver disease
NASH: Non-alcoholic steatohepatosis
ROHHAD: Rapid-onset obesity with hypothalamic dysfunction, hypoventilation, and autonomic dysregulation
ROHHADNET: Rapid-onset obesity, hypothalamic dysfunction, hypoventilation, autonomic dysregulation, and neuroendocrine tumor
