# Supplementary material for: Natural history of ROHHAD syndrome: development of severe insulin resistance and fatty liver disease over time
Source: Clin Diabetes Endocrinol. 2019 Jul 9;5:9. doi: 10.1186/s40842-019-0082-y (PMC6617654; doi:10.1186/s40842-019-0082-y)
Supplement: Supplementary file 1 — Table S1. Autonomic testing available on subject. Table S2 Summary data abstracted from reported cases and respective references. (DOCX 79 kb) [file 40842_2019_82_MOESM1_ESM.docx]

| **Table 1. Autonomic testing available on subject** |  |  |
| --- | --- | --- |
| Test* | Results | Treatment |
| Holter recording | Longest R-R 1.3 sec  Corrected QT (QTc) max 542 msec  HR 50-162 bpm | None required |
| Cardiac Autonomic testing | Valsalva HR 2.06 (43rd percentile for age)  Unable to perform deep breathing | None required |
| Tilt table testing | Impaired response to tilt-table test and to isoproterenol infusion, no syncope | Careful repositioning |
| Stationary bicycle testing | Not able to reach maximum effort | None required |
| Sleep study | Long central apneas and hypoventilation while breathing spontaneously during sleep as evidenced by decreased hemoglobin saturation, reiterating her ventilator dependence during sleep and her ETCO2 increased rapidly | Ventilator overnight with O2 supplementation, and oscilator, heart monitor |

*All tests were completed by age 16

| **Table 2. Summary data abstracted from reported cases and respective references** | | | | | | | | | | | | |
| --- | --- | --- | --- | --- | --- | --- | --- | --- | --- | --- | --- | --- |
|  |  |  |  | | **Sex** | |  |  |  |  |  |  |
| **N** | **First Author** | **N of cases** | | **Mean age at diagnosis** | **F** | **M** | **Year published** | **Hyper-**  **cholesterolemia** | **Insulin resistance** | **# reported deaths** | **Cause of death** | **Age at report (Yr)** |
| 1 | Lee(1) | - | | - | - | - | 2018 | - | - | - | - | - |
| 2 | Benvenga (2) | - | | - | - | - | 2018 | - | - | - | - | - |
| 3 | [Barclay](https://www.ncbi.nlm.nih.gov/pubmed/?term=Barclay%20SF%5BAuthor%5D&cauthor=true&cauthor_uid=30029683)(3) | - | | - | - | - | 2018 | - | - | - | - | - |
| 4 | Esparza(4) | 1 | | - | - | 1 | 2018 | - | - | - | - | 10.0 |
| 5 | Şiraz(5) | 2 | | - | 2 | - | 2018 | - | 1 | - | - | 6.2 |
| 6 | Lim(6) | 1 | | 2.4 | 1 | - | 2018 | - | - | - | - | 2.4 |
| 7 | Mitchell(7) | - | | 4.7 | - | - | 2017 | - | - | - | - | - |
| 8 | Pranzatelli(8) | - | | - | - | - | 2017 | - | - | - | - | - |
| 9 | Ibáñez-Micó(9) | 1 | | 3.5 | 1 | - | 2017 | - | - | 1 | Respiratory Pathology | - |
| 10 | Aljabban(10) | 1 | | 4.2 | 1 | - | 2016 | - | - | 1 | CPA | 4.7 |
| 11 | Al-Harbi(11) | 1 | | 5 | 1 | - | 2016 | - | - | - | - | 7.5 |
| 12 | Graziani(12) | 1 | | - |  | 1 | 2016 | - | - | - | - | 22.0 |
| 13 | Reppucci(13) | 6 | | - | 5 | 1 | 2016 | - | - | 1 | CPA | 14.7 |
| 14 | Jacobson(14) | 2 | | 2.3 | 1 | 1 | 2016 | - | - | - | - | 7.3 |
| 15 | Han(15) | - | | - | - | - | 2016 | - | - | - | - | - |
| 16 | Sanklecha(16) | 1 | | 2 | 1 | - | 2016 | - | - | 1 | CPA | - |
| 17 | Kot(17) | 1 | | 4 | - | 1 | 2016 | - | - | - | - | - |
| 18 | Barclay(18) | 16 | | 15 | - | - | 2016 | - | - | - | - | - |
| 19 | Cemeroglu (19) | 1 | | 12 | 1 | - | 2016 | - | - | - | - | - |
| 20 | Erensoy(20) | 1 | | 7 | 1 | - | 2016 | - | - | - | - | 8.0 |
| 21 | Maksoud(21) | - | | 6 | 1 | - | 2015 | - | - | - | - | 6.0 |
| 22 | Barclay(22) | 7 | | - | - | - | 2015 | - | - | - | - | - |
| 23 | Thaker(23) | 1 | | 8 | - | 1 | 2015 | 1 | 1 | - | - | - |
| 24 | Carroll(24) | 7 | | - | 4 | 3 | 2015 | - | - | - | - | 7.6 |
| 25 | Chow(25) | 1 | | - | - | 1 | 2015 | - | - | - | - | 15.0 |
| 26 | Benson(26) | - | | - | - | - | 2014 | - | - | - | - | - |
| 27 | Kocaay(27) | 1 | | 3 | 1 | - | 2014 | - | 1 | - | - | 13.0 |
| 28 | Patwari(28) | - | | - | - | - | 2014 | - | - | - | - | - |
| 29 | Cielo(29) | - | | - | - | - | 2014 | - | - | - | - | - |
| 30 | Sartori(30) | 2 | | 4.5 | 1 | 1 | 2014 | - | - | - | - | 4.5 |
| 31 | Sethi(31) | 1 | | - | 1 | - | 2014 | - | - | 1 | Septic shock | 5.0 |
| 32 | Ramistella(32) | 2 | | 1.5 | 2 | - | 2013 | - |  | - | - | 7.0 |
| 33 | Weese-Mayer(33) | - | | 4 | - | - | 2013 | - | - | - | - | 14.0 |
| 34 | Grudnikoff(34) | 1 | | 4 | 1 | - | 2013 | - | - | - | - | 14.0 |
| 35 | Dhondt(35) | 1 | | 7 | 1 | - | 2013 | - | - | 1 | CPA | 7.5 |
| 36 | Abaci(36) | - | | - | 1 | 1 | 2013 | - | - | - | - | 2.2 |
| 37 | Uçar(1)* | 1 | | - | 1 | - | 2013 | - | - | - | - | - |
| 38 | Chandrakantan(37) | 2 | | 5.4 | 2 | - | 2013 | - | 1 | - | - | 6.5 |
| 39 | Luccoli(38) | 1 | | - | 1 | - | 2012 | - | - | - | - | 3.0 |
| 40 | Armangue(39) | - | | - |  | - | 2012 | - | - | - | - | - |
| 41 | Sumanasena(40) | 1 | | 9.5 | 1 | - | 2012 | - | - | 1 | Pneumonia | 10.0 |
| 42 | Patwari(41) | 1 | | - | 1 | - | 2011 | - | - | - | - | - |
| 43 | Rand(42) | 25 | | - | - | - | 2011 | - | - | - | - | - |
| 44 | Chew(43) | 1 | | - | - | 1 | 2011 | - | - | - | - | 11.0 |
| 45 | Park(1)* | 1 | | - | - | 1 | 2010 | - | - | - | - | - |
| 46 | Carroll(44) | - | | - | - | - | 2010 | - | - | - | - | - |
| 47 | Lesser(45) | - | | - | - | - | 2009 | - | - | - | - | - |
| 48 | Bougnères(46) | 6 | | 19.6 | 9 | 6 | 2008 | - | - | - | - | - |
| 49 | Ize-Ludlow(47) | 15 | | - | - |  | 2007 | 3 | 1 | 1 | DC ventilatory support | - |
| 50 | Fishman(48) | 1 | | 3.7 | - | 1 | 1965 | - | - | - | - | 3.5 |
| CPA: cardiopulmonary arrest. * These two cases were mentioned in Reference 1, but actual detailed publications outside this reference could not be found. Note that liver disease is not included in this Table as we could not find mention of liver disease except a case in Reference 21 who was mentioned to have hepatitis C virus infection. None of the other cases documented nonalcoholic fatty liver disease, hepatic adenomas or hepatocellular cancer. | | | | | | | | | | | | |

**References**

1. Lee JM, Shin J, Kim S, Gee HY, Lee JS, Cha DH, et al. Rapid-Onset Obesity with Hypoventilation, Hypothalamic, Autonomic Dysregulation, and Neuroendocrine Tumors (ROHHADNET) Syndrome: A Systematic Review. Biomed Res Int. 2018;2018:1250721.

2. Benvenga S, Klose M, Vita R, Feldt-Rasmussen U. Less known aspects of central hypothyroidism: Part 2 - Congenital etiologies. J Clin Transl Endocrinol. 2018;14:5-11.

3. Barclay SF, Rand CM, Nguyen L, Wilson RJA, Wevrick R, Gibson WT, et al. ROHHAD and Prader-Willi syndrome (PWS): clinical and genetic comparison. Orphanet J Rare Dis. 2018;13(1):124.

4. Esparza Isasa E, Palomero Rodriguez MA, Acebedo Bambaren I, Medrano Vinas C, Gil Mayo D, Dominguez Perez F, et al. Anesthesia in a pediatric patient with ROHADD syndrome. Rev Esp Anestesiol Reanim. 2018;65(9):525-9.

5. Siraz UG, Okdemir D, Direk G, Akin L, Hatipoglu N, Kendirci M, et al. ROHHAD Syndrome, a Rare Cause of Hypothalamic Obesity: Report of Two Cases. J Clin Res Pediatr Endocrinol. 2018;10(4):382-6.

6. Lim SH, Jeon SY, Jeon IS, Kang MH. Rapid-onset obesity with hypothalamic dysfunction, hypoventilation, and autonomic dysregulation associated with neuroblastoma. Pediatr Blood Cancer. 2018;65(6):e26983.

7. Mitchell WG, Blaes F. Cancer and Autoimmunity: Paraneoplastic Neurological Disorders Associated With Neuroblastic Tumors. Semin Pediatr Neurol. 2017;24(3):180-8.

8. Pranzatelli MR, Tate ED, McGee NR. Demographic, Clinical, and Immunologic Features of 389 Children with Opsoclonus-Myoclonus Syndrome: A Cross-sectional Study. Front Neurol. 2017;8:468.

9. Ibanez-Mico S, Marcos Oltra AM, de Murcia Lemauviel S, Ruiz Pruneda R, Martinez Ferrandez C, Domingo Jimenez R. Rapid-onset obesity with hypothalamic dysregulation, hypoventilation, and autonomic dysregulation (ROHHAD syndrome): A case report and literature review. Neurologia. 2017;32(9):616-22.

10. Aljabban L, Kassab L, Bakoura NA, Alsalka MF, Maksoud I. Rapid-onset obesity, hypoventilation, hypothalamic dysfunction, autonomic dysregulation and neuroendocrine tumor syndrome with a homogenous enlargement of the pituitary gland: a case report. J Med Case Rep. 2016;10(1):328.

11. Al-Harbi AS, Al-Shamrani A, Al-Shawwa BA. Rapid-onset obesity, hypothalamic dysfunction, hypoventilation, and autonomic dysregulation in Saudi Arabia. Saudi Med J. 2016;37(11):1258-60.

12. Graziani A, Casalini P, Mirici-Cappa F, Pezzi G, Giuseppe Stefanini F. Hypoventilation improvement in an adult non-invasively ventilated patient with Rapid-onset Obesity with Hypothalamic Dysfunction Hypoventilation and Autonomic Dysregulation (ROHHAD). Pneumologia. 2016;65(4):222-4.

13. Reppucci D, Hamilton J, Yeh EA, Katz S, Al-Saleh S, Narang I. ROHHAD syndrome and evolution of sleep disordered breathing. Orphanet J Rare Dis. 2016;11(1):106.

14. Jacobson LA, Rane S, McReynolds LJ, Steppan DA, Chen AR, Paz-Priel I. Improved Behavior and Neuropsychological Function in Children With ROHHAD After High-Dose Cyclophosphamide. Pediatrics. 2016;138(1).

15. Han JC. Rare Syndromes and Common Variants of the Brain-Derived Neurotrophic Factor Gene in Human Obesity. Prog Mol Biol Transl Sci. 2016;140:75-95.

16. Sanklecha M, Sundaresan S, Udani V. ROHHAD Syndrome: The Girl who Forgets to Breathe. Indian Pediatr. 2016;53(4):343-4.

17. Kot K, Moszczynska E, Lecka-Ambroziak A, Migdal M, Szalecki M. ROHHAD in a 9-year-old boy - clinical case. Endokrynol Pol. 2016;67(2):226-31.

18. Barclay SF, Rand CM, Gray PA, Gibson WT, Wilson RJ, Berry-Kravis EM, et al. Absence of mutations in HCRT, HCRTR1 and HCRTR2 in patients with ROHHAD. Respir Physiol Neurobiol. 2016;221:59-63.

19. Cemeroglu AP, Eng DS, Most LA, Stalsonburg CM, Kleis L. Rapid-onset obesity with hypothalamic dysfunction, hypoventilation, and autonomic dysregulation syndrome and celiac disease in a 13-year-old girl: further evidence for autoimmunity? J Pediatr Endocrinol Metab. 2016;29(1):97-101.

20. Erensoy H, Ceylan ME, Evrensel A. Psychiatric Symptoms in Rapid-onset Obesity with Hypothalamic Dysfunction, Hypoventilation, and Autonomic Dysregulation Syndrome and its Treatment: A Case Report. Chin Med J (Engl). 2016;129(2):242-3.

21. Maksoud I, Kassab L. Rapid-onset obesity, hypoventilation, hypothalamic dysfunction, autonomic dysregulation syndrome. Avicenna J Med. 2015;5(3):89-94.

22. Barclay SF, Rand CM, Borch LA, Nguyen L, Gray PA, Gibson WT, et al. Rapid-Onset Obesity with Hypothalamic Dysfunction, Hypoventilation, and Autonomic Dysregulation (ROHHAD): exome sequencing of trios, monozygotic twins and tumours. Orphanet J Rare Dis. 2015;10:103.

23. Thaker VV, Esteves KM, Towne MC, Brownstein CA, James PM, Crowley L, et al. Whole exome sequencing identifies RAI1 mutation in a morbidly obese child diagnosed with ROHHAD syndrome. J Clin Endocrinol Metab. 2015;100(5):1723-30.

24. Carroll MS, Patwari PP, Kenny AS, Brogadir CD, Stewart TM, Weese-Mayer DE. Rapid-onset obesity with hypothalamic dysfunction, hypoventilation, and autonomic dysregulation (ROHHAD): Response to ventilatory challenges. Pediatr Pulmonol. 2015;50(12):1336-45.

25. Chow C, Fortier MV, Das L, Menon AP, Vasanwala R, Lam JC, et al. Rapid-onset obesity with hypothalamic dysfunction, hypoventilation, and autonomic dysregulation (ROHHAD) syndrome may have a hypothalamus-periaqueductal gray localization. Pediatr Neurol. 2015;52(5):521-5.

26. Benson LA, Olson H, Gorman MP. Evaluation and treatment of autoimmune neurologic disorders in the pediatric intensive care unit. Semin Pediatr Neurol. 2014;21(4):284-90.

27. Kocaay P, Siklar Z, Camtosun E, Kendirli T, Berberoglu M. ROHHAD Syndrome: Reasons for Diagnostic Difficulties in Obesity. J Clin Res Pediatr Endocrinol. 2014;6(4):254-7.

28. Patwari PP, Wolfe LF. Rapid-onset obesity with hypothalamic dysfunction, hypoventilation, and autonomic dysregulation: review and update. Curr Opin Pediatr. 2014;26(4):487-92.

29. Cielo C, Marcus CL. Central Hypoventilation Syndromes. Sleep Med Clin. 2014;9(1):105-18.

30. Sartori S, Priante E, Pettenazzo A, Marson P, Suppiej A, Benini F, et al. Intrathecal synthesis of oligoclonal bands in rapid-onset obesity with hypothalamic dysfunction, hypoventilation, and autonomic dysregulation syndrome: new evidence supporting immunological pathogenesis. J Child Neurol. 2014;29(3):421-5.

31. Sethi K, Lee YH, Daugherty LE, Hinkle A, Johnson MD, Katzman PJ, et al. ROHHADNET syndrome presenting as major behavioral changes in a 5-year-old obese girl. Pediatrics. 2014;134(2):e586-9.

32. Ramistella V, Wasniewska M, Valenzise M, Corica D, Cantucci S, Pitrolo E, et al. [A not very essential obesity: the Rohhad syndrome. Description of two cases and review of the literature]. Pediatr Med Chir. 2013;35(4):187-90.

33. Weese-Mayer DE, Rand CM, Ize-Ludlow D. Commentary: Rapid-onset Obesity with Hypothalamic Dysfunction, Hypoventilation, and Autonomic Dysregulation (ROHHAD): Remember Your ABCs (Airway, Breathing, Circulation). J Can Acad Child Adolesc Psychiatry. 2013;22(3):238-9.

34. Grudnikoff E, Foley C, Poole C, Theodosiadis E. Nocturnal Anxiety in a Youth with Rapid-onset Obesity, Hypothalamic Dysfunction, Hypoventilation, and Autonomic Dysregulation (ROHHAD). J Can Acad Child Adolesc Psychiatry. 2013;22(3):235-7.

35. Dhondt K, Verloo P, Verhelst H, Van Coster R, Overeem S. Hypocretin-1 deficiency in a girl with ROHHAD syndrome. Pediatrics. 2013;132(3):e788-92.

36. Abaci A, Catli G, Bayram E, Koroglu T, Olgun HN, Mutafoglu K, et al. A case of rapid-onset obesity with hypothalamic dysfunction, hypoventilation, autonomic dysregulation, and neural crest tumor: ROHHADNET syndrome. Endocr Pract. 2013;19(1):e12-6.

37. Chandrakantan A, Poulton TJ. Anesthetic considerations for rapid-onset obesity, hypoventilation, hypothalamic dysfunction, and autonomic dysfunction (ROHHAD) syndrome in children. Paediatr Anaesth. 2013;23(1):28-32.

38. Luccoli L, Ellena M, Esposito I, Bignamini E, Gregoretti C. Noninvasive ventilation in a child with hypothalamic dysfunction, hypoventilation, and autonomic dysregulation (ROHHAD). Minerva Anestesiol. 2012;78(10):1171-2.

39. Armangue T, Petit-Pedrol M, Dalmau J. Autoimmune encephalitis in children. J Child Neurol. 2012;27(11):1460-9.

40. Sumanasena SP, de Silva S, Perera I, Sudeen A, Wasala R. Rapid onset obesity, hypoventilation, hypothalamic, autonomic and thermal dysregulation, and neural tumour (ROHHADNET) syndrome presenting with Cushing syndrome. Ceylon Med J. 2012;57(1):47-8.

41. Patwari PP, Rand CM, Berry-Kravis EM, Ize-Ludlow D, Weese-Mayer DE. Monozygotic twins discordant for ROHHAD phenotype. Pediatrics. 2011;128(3):e711-5.

42. Rand CM, Patwari PP, Rodikova EA, Zhou L, Berry-Kravis EM, Wilson RJ, et al. Rapid-onset obesity with hypothalamic dysfunction, hypoventilation, and autonomic dysregulation: analysis of hypothalamic and autonomic candidate genes. Pediatr Res. 2011;70(4):375-8.

43. Chew HB, Ngu LH, Keng WT. Rapid-onset obesity with hypothalamic dysfunction, hypoventilation and autonomic dysregulation (ROHHAD): a case with additional features and review of the literature. BMJ Case Rep. 2011;2011.

44. Carroll MS, Patwari PP, Weese-Mayer DE. Carbon dioxide chemoreception and hypoventilation syndromes with autonomic dysregulation. J Appl Physiol (1985). 2010;108(4):979-88.

45. Lesser DJ, Ward SL, Kun SS, Keens TG. Congenital hypoventilation syndromes. Semin Respir Crit Care Med. 2009;30(3):339-47.

46. Bougneres P, Pantalone L, Linglart A, Rothenbuhler A, Le Stunff C. Endocrine manifestations of the rapid-onset obesity with hypoventilation, hypothalamic, autonomic dysregulation, and neural tumor syndrome in childhood. J Clin Endocrinol Metab. 2008;93(10):3971-80.

47. Ize-Ludlow D, Gray JA, Sperling MA, Berry-Kravis EM, Milunsky JM, Farooqi IS, et al. Rapid-onset obesity with hypothalamic dysfunction, hypoventilation, and autonomic dysregulation presenting in childhood. Pediatrics. 2007;120(1):e179-88.

48. Fishman LS, Samson JH, Sperling DR. Primary Alveolar Hypoventilation Syndrome (Ondine's Curse). Am J Dis Child. 1965;110:155-61.
